# Knockdown of Fibromodulin Inhibits Proliferation and Migration of RPE Cell via the VEGFR2-AKT Pathway

**DOI:** 10.1155/2018/5708537

**Published:** 2018-09-12

**Authors:** He Hu, Shanshan Li, Jianqiao Li, Chao Huang, Fang Zhou, Li Zhao, Wenzhen Yu, Xiao Qin

**Affiliations:** ^1^Medical College, Inner Mongolia University for the Nationalities, Tongliao, Inner Mongolia, China; ^2^Department of Ophthalmology, Qilu Hospital of Shandong University, Jinan, Shandong, China; ^3^Department of Blood Transfusion, Qilu Hospital of Shandong University, Jinan, Shandong, China; ^4^Department of Ophthalmology, Peking University People's Hospital, Key Laboratory of Vision Loss and Restoration, Ministry of Education, Beijing Key Laboratory of Diagnosis and Therapy of Retinal and Choroid Diseases, Beijing, China; ^5^Department of Ophthalmology, Affiliated Hospital of Inner Mongolia University for the Nationalities, Tongliao, Inner Mongolia, China

## Abstract

**Purpose:**

Recent research has provided novel insight into the function of fibromodulin (FMOD) in wound healing and angiogenesis. The role of FMOD in initiation of proliferative vitreoretinopathy (PVR) has not been studied. This study investigated the effect of FMOD on human retinal pigment epithelial (RPE) cell, which plays an essential role in the progression of PVR, and the possible mechanisms.

**Methods:**

Small interfering (si) RNA-based gene transfer technology was used to decrease FMOD expression and to study its effects on RPEs *in vitro*. Cell Counting Kit-8 assays, transwells, and flow cytometry analysis were used to measure cell proliferation, migration, cell cycle, and apoptosis. Western blot was used to measure expression of vascular endothelial growth factor (VEGF), VEGF receptor 2 (VEGFR2), extracellular signal-related kinase 1/2 (ERK1/2), and phosphoinositide 3 kinase (PI3K/AKT).

**Results:**

After transfection of RPEs with a FMOD-specific siRNA, cell proliferation and migration were inhibited to the percentage of 65% ± 5% and 39% ± 10%, respectively, compared to the control group. Depletion of FMOD induced cell cycle arrest and apoptosis in RPE cells. Downregulation of VEGF, VEGFR2, and phosphorylated AKT (p-AKT) were detected in transfected RPEs.

**Conclusion:**

Depletion of FMOD selectively downregulated the expression of VEGF and VEGFR2 and inhibited the signaling pathway of AKT phosphorylation, which consequently inhibited the proliferation and migration of RPE Cell.

## 1. Introduction

The retinal pigment epithelial (RPE) is a monolayer of hexagonally packed cells, situated between the choroid and neural retina in the eye [1–3]. In normal physiological states, RPE is connected by tight-junctions, which constitutes the outer blood-retinal barrier (BRB) [1]. RPE also plays important roles in maintaining ocular physiological conditions, such as in absorption of light [4], maintenance of the visual cycle [5], phagocytosis of shed photoreceptor, transepithelial transport [1], immune privilege of the eye, and the secretion of many essential factors [6]. However, under pathological conditions, caused by the changes of internal and external environment, RPE cells are correlated with the initiation and development of various ophthalmic diseases. It has been indicated that RPE contributes to the development of fibroproliferative ocular disease [3, 7–11]. Exploring the upstream regulator and molecular mechanism of RPE activation is an important topic in the study of ocular diseases.

FMOD, a secreted protein, is a member of the small leucine-rich proteoglycan (SLRP) family. It is mainly expressed in mesenchymal connective tissue and involved in a series of biological and pathophysiological processes [12, 13]. FMOD was reported to play important roles in modulating extracellular matrix (ECM) organization [14] and collagen fibrillogenesis [15, 16] and also is reported as a regulator of cell reprogramming [17, 18]. However, the role that FMOD played in RPEs, which strongly associated with fibroproliferative ocular diseases, has not been documented before.

In this study, we provide insights into the effects and possible mechanisms of FMOD on RPE behavior for the ﬁrst time using a small interfering (Si) RNA. According to our results, FMOD is critical to migration and proliferation of RPE. We demonstrate that depletion of FMOD inhibited proliferation and migration of RPE through selective downregulation of vascular endothelial growth factor (VEGF) and VEGF receptor 2 (VEGFR2) expressions, which leads to inhibition of the phosphoinositide 3 kinase (PI3K/AKT) phosphorylation. These promising results suggest FMOD as a potential therapeutic target for RPE-related ocular diseases.

## 2. Methods

### 2.1. Cell Culture and siRNA Transfection

We obtained RPE cells (ARPE19 cell line, CRL-2302, VA, USA) from the American Type Culture Collection (ATCC, Manassas, VA, USA). The cells were cultured in Dulbecco's modiﬁed Eagle's medium (DMEM)/F12 medium (HyClone, Grand Island, NY, USA) with 10% fetal bovine serum (FBS; Gibco, Invitrogen, Grand Island, NY, USA) at 37°C in 5% CO_2_ and 95% humidity, as recommended by ATCC. The cells were plated in six-well culture dishes and were used for experiments at 60% to 80% conﬂuence. The RPE cells were transfected with fibromodulin-specific small interfering (si) RNAs (FMOD-siRNA, Cat#sc-40995; Santa Cruz Biotechnology, Inc., California, USA). Transfections were performed using the Lipofectamine® 2000 transfection reagent (Cat#11668019; Invitrogen) according to the manufacturer's protocol. Nonsilencing siRNA (NS-siRNA, Cat#sc-37007; Santa Cruz Biotechnology, Inc.) was used to control for any effect of siRNA and the transfection reagent. Following assays were performed 48 h after transfection, including assessment of cell proliferation, cell cycle, apoptosis, migration, and western blot analysis.

### 2.2. Cell Proliferation Assay

The cell proliferation assay was performed as described previously [19]. The RPE cells were incubated in 96-well plates. Cell Counting Kit-8 (CCK-8; Dojindo, Shanghai, China) assays were performed according to the manufacturer's instructions and read using an ELISA microplate reader (Finstruments Multiskan Models 347; MTX Lab Systems, Inc., Vienna, VA, USA). Each experiment was performed in ﬁve wells and repeated at least three times.

### 2.3. Flow Cytometry Analysis of Cell Apoptosis and Cell Cycle

A cell apoptosis study (FITC Annexin V Apoptosis Detection Kit; BD Science) and cell cycle analysis (Cycletest Plus DNA Reagent Kit; BD Science) were performed according to the manufacturer's instructions. Briefly, RPE cells (1 × 10^6^) were seeded in 6-well plates and transfected with NS-siRNA and FMOD-siRNA for 48 h. The samples were analyzed using flow cytometry (FACSCalibur; BD Biosciences); the experiments were performed in triplicate and repeated at least three times. The apoptotic rate was calculated by the percentage of early apoptotic cells (LR) added with late apoptotic cells (UR).

### 2.4. Cell Migration Assay

The RPE cells migration study was performed using Transwells (Cat#3422; Corning, Tewksbury, MA, USA) as described previously [19]. Brieﬂy, 5 × 10^4^ cells in 200 *μ*L serum-free medium were placed in the upper portion of a Transwell. The DMEM/F12 medium (containing 10% FBS) was placed in the bottom chamber at a ﬁnal volume of 600 *μ*L. All migration assays were conducted at 37°C for 6 hours, and the cells were then ﬁxed in 4% paraformaldehyde (PFA) and stained with 4′,6-diamidino-2-phenylindole (DAPI; Roche Diagnostics, Indianapolis, IN, USA). The cells that had not migrated through the membrane were removed with a cotton swab, and the membrane was imaged with ﬂuorescence microscopy (Zeiss Axiophot, Thornwood, NY, USA). The RPE cells from ﬁve random ﬁelds of view were counted. Each experiment was repeated at least three times.

### 2.5. Western Blot Analysis

RPE cells were prepared with protein extraction and protease inhibitor kits (Pierce, Rockford, IL, USA). After centrifugation, the supernatant was collected, and the protein lysate was measured with a bicinchoninic acid (BCA) protein assay kit (Pierce) according to the manufacturer's instructions. Equal amounts of protein were separated by 12% SDS-PAGE and transferred electrophoretically to nitrocellulose membranes (Amersham, Little Chalfont, UK). The proteins were visualized with enhanced chemiluminescence Western blot (W-B) detection reagents (Pierce). Band densities were tested with antibodies against FMOD (1 : 200, sc-33772; Santa Cruz Biotechnology, Inc.), VEGF (1 : 200, ab46154; Abcam, Cambridge, MA, USA), VEGFR2 (1 : 1000, #55B11; Cell Signaling Technology (CST), Danvers, MA, USA), phosphoinositide 3 kinase (PI3K/AKT) (1 : 1000, #4685; CST), phosphorylated AKT (p-AKT) (1 : 1000, #4060; CST), ERK1/2 (1 : 1000, #9102; CST), and phosphorylated ERK1/2 (p-ERK1/2) (1 : 1000, #4370; CST), followed by incubation with a horseradish peroxidase (HRP)-conjugated goat antibody against rabbit IgG (1 : 1000, #7074; CST). For sequential blotting with additional antibodies, the membranes were stripped with a restorative western blot stripping buffer and reprobed with the indicated antibodies. Western blot analyses were repeated at least three times, and qualitatively similar results were obtained.

### 2.6. Statistical Analysis

Data analysis was performed using the statistical software Prism 5 (GraphPad Software, Inc., San Diego, CA, USA). All data are presented as the means ± SEM. Differences between experimental and control groups were made using Student's *t*-test. A *P* value of less than 0.05 was considered to be statistically signiﬁcant.

## 3. Results

### 3.1. siRNA Knocked Down the Expression of FMOD in RPE Cells

**Figure 1:**
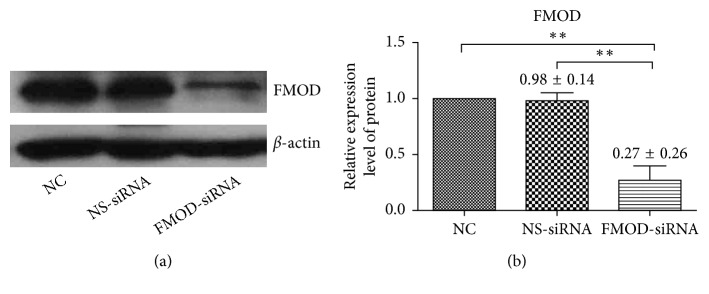
Expression of FMOD in RPE cells. FMOD expression in human RPE cells was significantly knocked down in FMOD-siRNA-treated group at the protein level, as measured by western blot 48 h after transfection. There is no significant difference in normal control (NC) and nonsilencing siRNA-treated (NS-siRNA) RPE cells. The expression of FMOD in NC group was set to 100%: (a) representative blot images; (b) statistical analysis of FMOD expression. Data are represented as the means ± SEM of fold changes compared to the controls. Each experiment was repeated at least three times. ^*∗∗*^*P* < 0.01.

Western blot analyses were performed to detect the effectiveness of FMOD-siRNA on RPE cells. As shown in Figure 1, FMOD-siRNA effectively reduced FMOD protein levels in RPE cells (*P* < 0.01). There was no significant difference between the nonsilencing siRNA-treated (NS) RPE cells and normal control (NC) ones (*P* > 0.05). These results demonstrated that siRNA successfully knocked down expression of FMOD in RPE cells.

### 3.2. Depletion of FMOD Inhibited Proliferation and Migration in RPE

**Figure 2:**
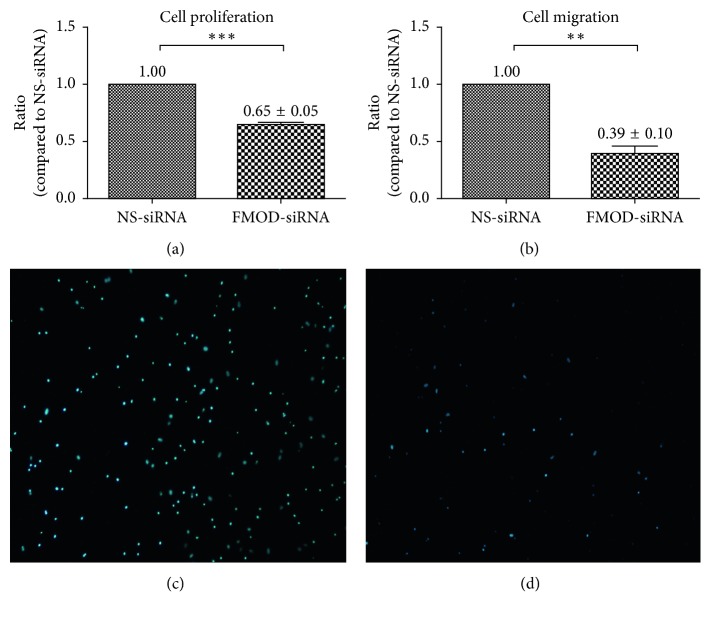
Effects of FMOD on proliferation and migration in RPE cells. The results of the CCK-8 proliferation assay revealed that FMOD-siRNA-treated RPE cells displayed decreased proliferation compared with the control group: (a) statistical analysis of CCK-8 proliferation assay; statistical analysis of the Boyden Chamber assay revealed that the number of cells that passed through the membrane in the FMOD-siRNA group was significantly lower than the number in the control group: (b) statistical analysis of Boyden Chamber assay; (c) NS-siRNA-treated RPE cells; and (d) FMOD-siRNA-treated RPE cells. Data are presented as the means ± SEM. Each experiment was repeated at least three times. ^*∗∗*^*P* < 0.01; ^*∗∗∗*^*P* < 0.001.

We performed experiments to evaluate whether inhibition of FMOD expression had any effect on aspects of the biological activities of RPE cells. A CCK-8 Proliferation Assay Kit was used to evaluate the effects of depletion FMOD on RPE cells, and the results revealed that RPE cells transfected with the FMOD-siRNA decreased proliferation compared with the NS-siRNA group (Figure 2(a), *P* < 0.01). The Boyden Chamber assay was used to evaluate effect of FMOD on RPE cell migration. As shown in Figures 2(b)–2(d), the number of FMOD-siRNA-treated cells that passed through the membrane was significantly lower than the number of control cells. These results suggested that knockdown of FMOD effectively inhibited biological activity in RPE cells (*P* < 0.01).

### 3.3. Depletion of FMOD Induced Cell Cycle Arrest and Apoptosis in RPE Cells

**Figure 3:**
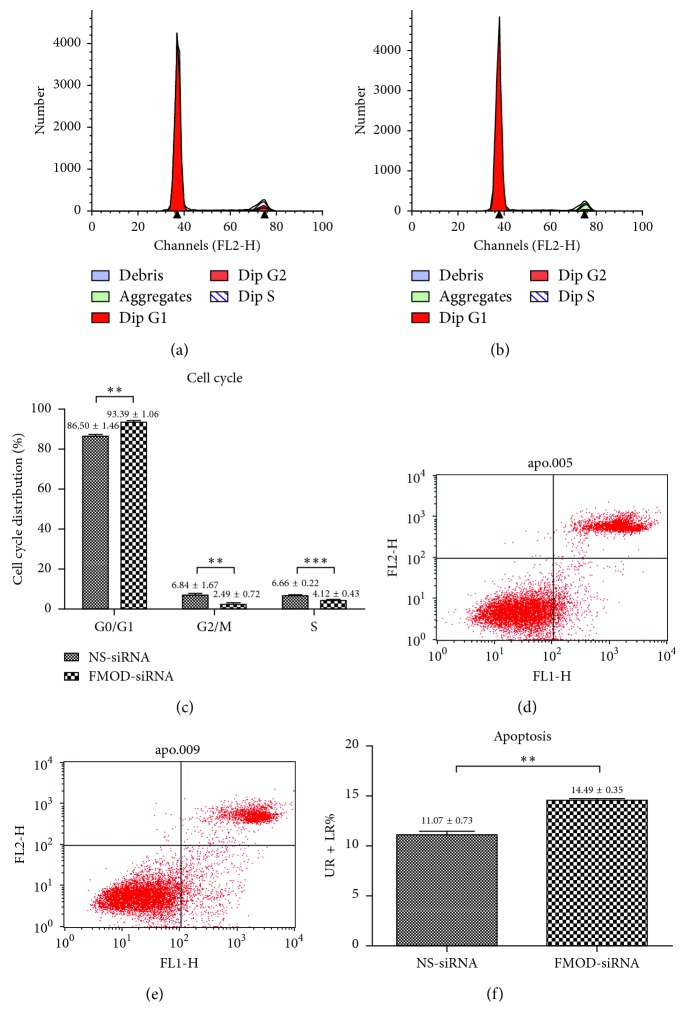
Effects of FMOD on the cell cycle and cell apoptosis in RPE cells. The results of the flow cytometry analysis revealed that FMOD-siRNA-treated RPE cells accumulated more in the G0/G1 phase but less in the G2/M and the S phase of the cell cycle compared with the NS-siRNA group: (a) NS-siRNA-treated RPE cells; (b) FMOD-siRNA-treated RPE cells; (c) statistical analysis of the cell cycle. The percentage of early apoptotic cells plus late apoptotic cells in the FMOD-siRNA-treated group was significantly lower than in the controls, indicating that FMOD depletion induced cell apoptosis in RPE cells: (d) NS-siRNA-treated RPE cells; (e) FMOD-siRNA-treated RPE cells; (f) statistical analysis of cell apoptosis. Data are presented as the means ± SEM. Each experiment was repeated at least three times. ^*∗∗*^*P* < 0.01; ^*∗∗∗*^*P* < 0.0001.

Flow cytometry analysis was performed to detect the cell cycle and apoptotic rate in RPE cells. As shown in Figures 3(a)–3(c), FMOD-siRNA-treated RPE cells accumulated more in the G0/G1 phase but less in the G2/M and the S phase of the cell cycle compared with the NS-siRNA group (*P* < 0.05). As shown in Figures 3(d)–3(f), the apoptotic rate, which was calculated by the percentage of early apoptotic cells (LR) plus late apoptotic cells (UR), was significantly increased in the FMOD-siRNA-treated group than in the NS-siRNA one (*P* < 0.05). These results indicated that knockdown of FMOD induced cellular arrest and apoptosis in RPE cells.

### 3.4. Depletion of FMOD Downregulated Expression of VEGF and Inhibited VEGFR2-AKT Signaling Pathway in RPE Cells

**Figure 4:**
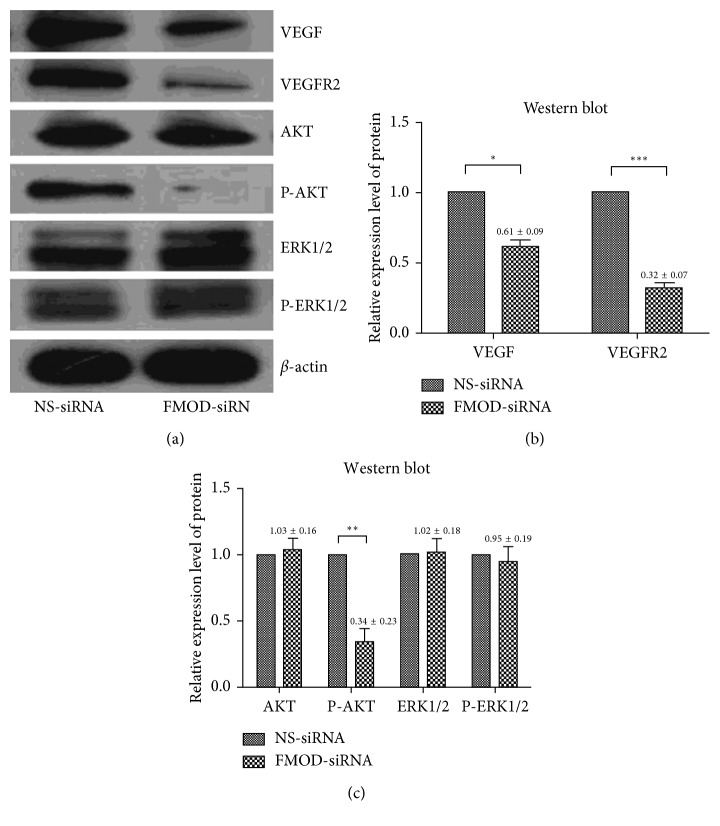
Western blot analysis of protein in RPE cells. The results of the western blot analysis revealed that, VEGF, VEGFR2, and p-AKT protein expression levels were significantly downregulated in the FMOD-siRNA group, while Akt, ERK1/2 and p-ERK1/2 expression levels did not differ between the two groups: (a) representative blot images; (b) and (c): statistical analysis of western blot data. Data are represented as the means ± SEM of fold changes compared to the controls. Each experiment was repeated at least three times. ^*∗*^*P* < 0.05; ^*∗∗*^*P* < 0.01; ^*∗∗∗*^*P* < 0.0001.

As mentioned, depletion of FMOD inhibited proliferation and migration in RPE cells and induced cell cycle arrest and apoptosis. To investigate a possible mechanism of FMOD on the biological activity of RPE cells, we performed western blot analyses to assess the respective protein levels of VEGF, VEGFR2, and the expression and phosphorylation levels of Akt and ERK1/2, with *β*-actin serving as an internal loading control (Figure 4). We observed significant downregulation of VEGF and VEGFR2 in FMOD-siRNA-treated RPE cells compared with the control group (*P* < 0.05). Proteins downstream of VEGFR2, such as AKT and ERK1/2, were detected, and only AKT phosphorylation was decreased (*P* < 0.05), whereas AKT and ERK1/2 expression and ERK1/2 phosphorylation remained at almost the same levels in the two groups (*P* > 0.05) (Figures 4(a)–4(c)).

## 4. Discussion

Evidence has indicated that RPE cells are strongly associated with the progression of PVR [20, 21]. Accompanied by the breaking of retinal integrity, RPE cells are exposed to the vitreous which is rich of growth factors/cytokines. The trauma also disturbs the quiescent RPE monolayer and physically displaces some of the RPE cells into the vitreous [3]. The cells are activated and undergo epithelial-mesenchymal transition (EMT), a process in which RPE loses epithelial characteristics, such as cell-to-cell contact, but acquire stronger abilities of migration, proliferation, antiapoptosis, and ECMdegradation, which are properties of mesenchyma [22–24]. Moreover, RPEs convert from epithelia into fibrotic cells and become the primary cause of the genesis of preretinal/epiretinal membrane [24]. The formation of this proliferative membrane is a critical pathological progression of PVR. Therefore, the abnormal conversion of RPE was considered to play an essential role in the pathogenesis of PVR [3, 7–11, 23].

Although most PVR cases can be repaired by modern surgical techniques, the visual recovery after operation is not promising because of the retinal damage caused by PVR. Likewise, how to design satisfactory therapeutic strategies for PVR is also a conundrum in clinics because the understanding of mechanisms and pathogenesis of PVR still remains incomprehensive. As a result, exploring the potential upstream regulator of RPE activation so as to find new targets for the treatment of PVR has undoubtedly become an urgent problem in PVR research.

In recent years, increasing studies have suggested FMOD as a crucial regulator of wound healing [12, 17, 25–28], which leads to the speculation that FMOD may also play a special role in the pathological progression of PVR, the process of wound healing in the eye. It is known that RPE plays critical roles in eye fibrosis, which raises the question that whether FMOD is associated with the abnormal activation of RPE. The question has not been documented before. Therefore, in our experiment, small interfering (Si) RNAs-based gene transfer technology was performed to inhibit FMOD expression in RPE cells, in order to evaluate the effects of depletion FMOD on biological activities of RPEs. Our results demonstrated that inhibition of FMOD expression had remarkable effect on RPE biological activities. More specifically, depletion of FMOD led to the decreased ability of proliferation and migration and also induced cell cycle arrest and apoptosis in RPE cells. These results indicated that FMOD may play important roles in PVR. However, this speculation still needs to be validated in further animal experiments.

EMT is integral in development, wound healing and stem cell behavior, and contributes pathologically to fibrosis and cancer progression [29]. Recent studies have demonstrated that EMT of RPE plays an essential role in the pathological progression of PVR. Multiple factors were proved to play roles in the progression of EMT [7–11, 22–24]. In physiological states, RPE secretes a variety of growth factors, including VEGF, pigment epithelium-derived factor (PEDF) [30], transforming growth factor-*β* (TGF-*β*), ciliary neurotrophic factor (CNTF), platelet-derived growth factor (PDGF), and tumor necrosis factor *a* (TNF-*α*) [31]. Among these factors, VEGF and TGF-*β* have been found in vitreous fluid of PVR patients and reported to be involved in EMT of RPE and pathogenesis of PVR [29, 32, 33]. VEGF is secreted in low concentration by RPE in healthy eyes, but overproducion in pathological state indicated that it may play an important role in pathological progression of RPE. In recent years, more attention has been paid on mechanisms and potential upstream regulators of RPE in ocular diseases.

In addition, to investigate a possible molecular mechanism of FMOD on the biological and angiogenic activity of RPE cells, we assessed the expression levels of relevant regulators, such as VEGFA, VEGFR2, which is the most important responser of VEGFA [34], and downstream effectors of VEGFR2, including PI3K/Akt and ERKs [35]. In our experiment, we observed significant downregulation of VEGFA and VEGFR2 in FMOD inhibited RPEs. AKT phosphorylation was also decreased, whereas AKT, ERK expression, and ERK phosphorylation remained at almost the same levels. The results proved that FMOD was involved in the regulation of VEGF-VEGFR2-AKT signaling pathway in RPE cells. Moreover, the inhibition of proliferation and migration of RPEs caused by the depletion of FMOD may be carried out by the downregulation of VEGF-VEGFR2 and the inhibition of AKT phosphorylation.

In conclusion, this is the first report to identify the involvement of FMOD in biological activity of RPEs and suggested that depletion of FMOD inhibited migration and proliferation of RPE through selective downregulation of VEGFR2 expression, which lead to inhibition of the AKT phosphorylation. FMOD performed functions in activity of RPE, and further studies are required to investigate the participation of FMOD in PVR, which may be important for the development of clinical therapeutic tools.

## Data Availability

The data used to support the findings of this study are included within the article.
